# House Insects

**Published:** 1874-10

**Authors:** 


### HOUSE INSECTS.

Ants, black and red, cockroaches, spiders, chintz bugs, and other crawling pests, may be got rid of thus: Dissolve two pounds of alum in a gallon of boiling water; let it remain on the fire until the alum disappears, then apply it, while boiling hot, with a brush to every crack and crevice from which they emanate; hence alum dissolved in whitewash makes a good vermin riddance. Cockroaches will avoid painted surfaces which have been washed with cold alum water. A well checked mark around a barrel or box will prevent their approach by insects. Powdered alum scattered over the pillows and sheets will keep away bed bugs for the night. If these things are so, it might be well for travelers to make a pint of pulverized alum a part of their outfit.

### MOTHS.

To half a pint each of alcohol and turpentine, add two ounces of gum camphor; keep in a glass or stone bottle, and shake before using; crumble up pieces of blotting paper, drop them in the mixture, and distribute among the clothing or furs; then wrap each article in a piece of Irish linen or untorn newspaper. This should be done during March in northern latitudes. If an article is well wrapped up in a newspaper, which is laid on the floor, and the wrapping commenced at one corner, so that two corners can be turned in, leaving only the fourth corner to be fastened with a pin or paste, and if the same thing be done with another paper, no camphor or anything else need be enfolded, because the paper being unpervious, more so than Irish linen, the moth would never deposit an egg, because both materials are too cold for the life of the insect, and paternal instinct points this out.

